# Preventing foot ulceration in diabetes: systematic review and meta-analyses of RCT data

**DOI:** 10.1007/s00125-019-05020-7

**Published:** 2019-11-27

**Authors:** Fay Crawford, Donald J. Nicolson, Aparna E. Amanna, Angela Martin, Saket Gupta, Graham P. Leese, Robert Heggie, Francesca M. Chappell, Heather H. McIntosh

**Affiliations:** 1 NHS Fife, Queen Margaret Hospital, Dunfermline, KY12 0SU UK; 2 School of Medicine, University of St Andrews, Fife, UK; 3 NHS Tayside, Dundee, UK; 4 Health Economics and Health Technology Assessment (HEHTA) Institute of Health and Wellbeing College of Medical, Veterinary and Life Sciences, University of Glasgow, Glasgow, UK; 5 The Centre for Clinical Brain Sciences (CCBS) Neuroimaging Sciences, University of Edinburgh, Edinburgh, UK; 6 Healthcare Improvement Scotland, Glasgow, UK

**Keywords:** Diabetes, Evidence-based healthcare, Foot ulcer, Meta-analysis, Prevention, Systematic review

## Abstract

**Aims/hypothesis:**

Foot ulceration is a serious complication for people with diabetes that results in high levels of morbidity for individuals and significant costs for health and social care systems. Nineteen systematic reviews of preventative interventions have been published, but none provides a reliable numerical summary of treatment effects. The aim of this study was to systematically review the evidence from RCTs and, where possible, conduct meta-analyses to make the best possible use of the currently available data.

**Methods:**

We conducted a systematic review and meta-analysis of RCTs of preventative interventions for foot ulceration. OVID MEDLINE and EMBASE were searched to February 2019 and the Cochrane Central Register of Controlled Trials to October 2018. RCTs of interventions to prevent foot ulcers in people with diabetes who were free from foot ulceration at trial entry were included. Two independent reviewers read the full-text articles and extracted data. The quality of trial reporting was assessed using the Cochrane Risk of Bias tool. The primary outcome of foot ulceration was summarised using pooled relative risks in meta-analyses.

**Results:**

Twenty-two RCTs of eight interventions were eligible for analysis. One trial of digital silicone devices (RR 0.07 [95% CI 0.01, 0.55]) and meta-analyses of dermal infrared thermometry (RR 0.41 [95% CI 0.19, 0.86]), complex interventions (RR 0.59 [95% CI 0.38, 0.90], and custom-made footwear and offloading insoles (RR 0.53 [95% CI 0.33, 0.85]) showed beneficial effects for these interventions.

**Conclusions/interpretation:**

Four interventions were identified as being effective in preventing foot ulcers in people with diabetes, but uncertainty remains about what works and who is most likely to benefit.

**Electronic supplementary material:**

The online version of this article (10.1007/s00125-019-05020-7) contains peer-reviewed but unedited supplementary material, which is available to authorised users.


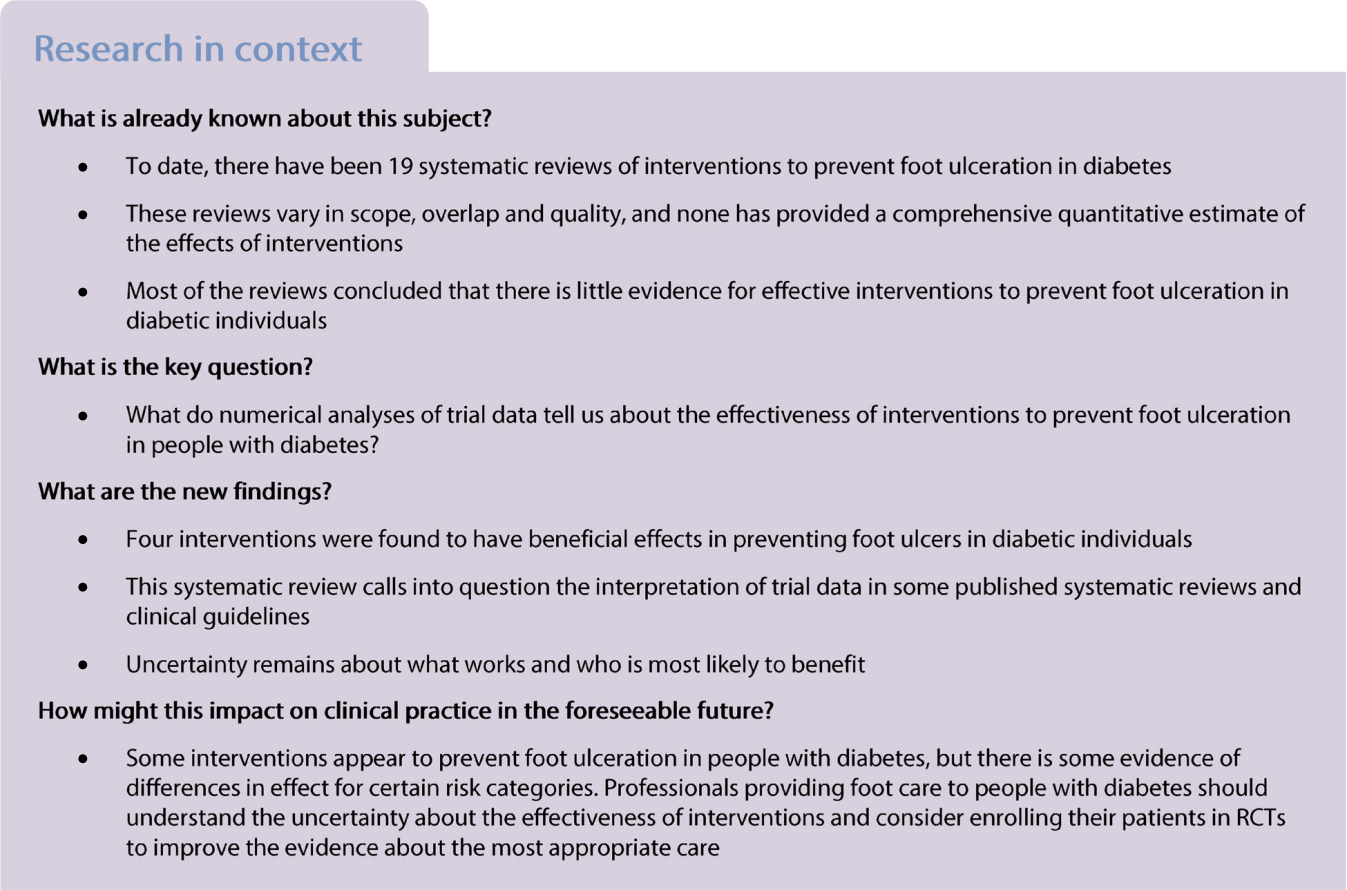


## Introduction

Foot ulceration is a serious complication of diabetes that can result in high levels of morbidity for individuals and burdens health and social care systems with huge costs [1, 2]. Predicting those people most likely to develop a foot ulcer has been the subject of much research and the independent risk factors have been established [3, 4]. However, the value of prediction models to inform treatment decisions depends on the availability of effective interventions to modify risk [5].

As part of a wider research project to create a cost-effective, evidence-based pathway for assessing and managing the foot in diabetes, we conducted an overview of existing systematic reviews to synthesise the available evidence on treatment effects (PROSPERO registration: CRD42016052324). Although the overview identified 19 published reviews [6–24], it failed to provide reliable numerical summaries of effects because of limitations of the reviews in scope, overlap and quality [25]. A comprehensive review of RCTs was required to enable us to make the best possible use of the data currently available and re-explore the possibility of performing meta-analyses.

## Methods

Our aim was to systematically review data from RCTs of interventions used to prevent foot ulcerations in diabetes, and to conduct meta-analyses to obtain pooled estimates of their effects. We included data from RCTs only, as this is the only method of clinical evaluation that controls for known, unknown and unmeasured confounding.

The protocol can be viewed at www.journalslibrary.nihr.ac.uk/programmes/hta/1517101.

### Eligibility criteria

Trials were permitted to include people of any age with a diagnosis of type 1 or type 2 diabetes, with or without a history of ulceration, but free from foot ulceration at trial entry.

Simple interventions (e.g. education aimed at individuals with diabetes or physicians, or the provision of footwear) and complex interventions (where several interventions were provided together) were eligible for inclusion. Standard care or active treatment were eligible as comparators.

### Outcomes

#### Primary outcomes

We were primarily interested in foot ulcers (incident, primary and recurrent) reported as binary outcomes (present/absent). These could be defined, for example, as ‘a full-thickness skin defect that requires more than 14 days to heal’ [26] or according to a system of ulcer classification [27]. Primary outcomes were the absolute numbers of incident primary ulcers and of incident recurrent ulcers.

#### Secondary outcomes

In reports where foot ulceration was the primary outcome we also sought data on amputation (minor: involving the foot [intrinsic to the foot]; or major: involving the foot and leg); mortality; gangrene; infection; adverse events; harms; time to ulceration; quality of life (measured using the EuroQol five-dimensions questionnaire or the six- or 12-item Short Forms); timing of screening; self-care; hospital admissions; psychological (knowledge/behaviour); and adherence to therapy.

### Searches

We searched OVID MEDLINE (see electronic supplementary material [ESM] Table 1) and OVID EMBASE (from inception to February 2019) and the Cochrane Central Register of Controlled Trials (to October 2018) for eligible RCTs, without language restrictions. ClinicalTrials.gov was searched for ongoing clinical trials (search date: 21 February 2019).

### Trial selection and data extraction

One reviewer screened all titles and abstracts and a 10% random sample was checked by a second reviewer. Two reviewers working independently screened full-text articles and extracted data (D. J. Nicholson, and either F. Crawford or A. E. Amanna) about the included populations, including the risk classification, interventions, comparators and outcomes. For each trial we extracted absolute numbers on an intention-to-treat basis, where the numbers randomised to each group were available, and calculated RRs and 95% CIs. Where reports lacked information or clarity, we contacted the trial authors. Non-English language reports were translated.

### Risk of bias (quality) assessment

We assessed the quality of trial reporting using the Cochrane Risk of Bias tool [28]. The five domains we assessed were: random sequence generation, allocation concealment, blinding of assessors to the outcome, incomplete outcome data and selective reporting [28]. We also noted whether an a priori sample size calculation was reported [29].

### Data analysis

Absolute numbers were extracted and RRs and 95% CIs were calculated. Where it made clinical and statistical sense to pool the data, we undertook meta-analyses with trial data weighted according to the inverse variance method and assessed heterogeneity using the *I*^2^ statistic [28]. Analyses were conducted using R version 3.4.2 (https://cran.r-project.org).

## Results

**Fig. 1:**
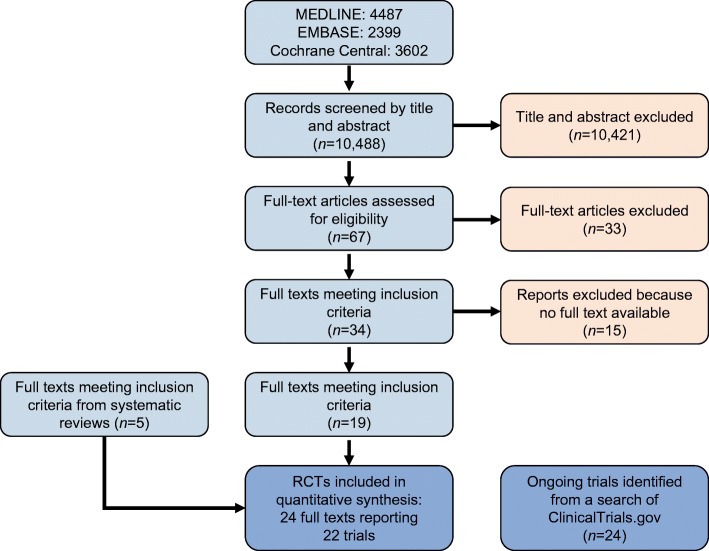
Flow diagram of study selection

From 10,488 studies, 22 RCTs met our eligibility criteria [30–51]. A flow diagram showing the flow of information throughout the process of screening and selecting studies for inclusion in the review is presented in Fig. 1 and the characteristics of the included trials are described in Table 1. Table 1 also incorporates the results from the risk of bias assessment; only five of the 22 trials [36, 39, 42, 46, 50] were judged to be at low risk of bias.

**Table 1:** Characteristics of included trials

Author	Population characteristics	Details of experimental and control interventions	Standard care	Outcomes (unit of analysis) Length of follow-up	Risk of bias^a^
Education					
Monami 2015 [34]	*n* = 121 (I = 61, C = 60) Male: 60% Mean age: 71 years Previous ulcers: 11% T2DM: 100% Mean diabetes duration: 15 years Ulcer risk: high Participants defined as ‘high risk’ if neuropathy diagnosed, previous diabetic foot ulcer or foot abnormalities	Intervention: brief educational programme 2 h programme provided by a physician (for 15 min) and nurse (for 105 min) to groups of five to seven participants: 30 min face-to-face lesson on risk factors for foot ulcers and 90 min interactive session with practical exercises on behaviours for reducing risk Control: brief leaflet and standard care	All participants had previously received standard multidisciplinary education for diabetes (with a structured group programme at diagnosis or first contact, and follow-up meetings every 2 years)	Ulcers (*n*), amputation (*n*), mortality (*n*), knowledge score, time spent for intervention and ulcer care in control group (min per participant) Follow-up: 6 months	Sequence generation: + Allocation concealment: + Assessor blinding to outcome data: – Incomplete data addressed: – Selective reporting: + Sample size calculated: +
Annersten Gershater 2011 [35]	*n* = 131 (I = 61, C = 70) Male: 73% Mean age: 64 years Previous ulcers: 100% T2DM: 67% Mean diabetes duration: NR Ulcer risk: high (IWGDF)	Intervention: group session of foot care education from a registered diabetes nurse Oral and written instructions on self-care based on IWGDF guidelines 1× 60 min plus standard care Control: standard information, oral and written instructions on self-care based on IWGDF guidelines	Routine care from staff Adjusted shoes for indoor and outdoor use and individually fitted insoles	Ulcers (*n*), cause of ulcers (stress, trauma, other), location of ulcers (big toe or other, plantar, other including heel) Follow-up: 6 months	Sequence generation: + Allocation concealment: + Assessor blinding to outcome data: – Incomplete data addressed: + Selective reporting: + Sample size calculated: +
Lincoln 2008 [36]	*n* = 172 (I = 87, C = 85) Male: 67% Mean age: NR Previous ulcers: 100% T2DM: 77% Mean diabetes duration: NR Ulcer risk: high (10 g monofilament, Neurotip, VPT ≥25 V)	Intervention: 1 h structured foot care education session provided by the researcher in participants’ own homes Control: standard care and the same foot care leaflets as the intervention group	Regular podiatry and suitable orthoses when appropriate Overall medical care followed national UK clinical guidelines	Ulcers (*n*), amputations (*n*), quality of life (DFS-SF), mood (HADS, HADS-anxiety, HADS-depression), protective foot care behaviours (NAFF) Follow-up: 6 and 12 months	Sequence generation: + Allocation concealment: + Assessor blinding to outcome data: + Incomplete data addressed: + Selective reporting: + Sample size calculated: +
Dermal infrared thermometry					
Armstrong 2007 [37]	*n* = 225 (I = NR, C = NR) Male: 96% Mean age: 69 years Previous ulcers: unclear T2DM: 100% Mean diabetes duration: 13 years Ulcer risk: IWGDF risk group 2/3	Intervention: infrared thermometry and a complex intervention provided by attending physicians Control: a complex intervention only	Footwear, education and professional foot care	Ulcers (*n*, %), rate of ulcer (HR), temperature difference at ulcer site (survival curve) Follow-up: 18 months	Sequence generation: + Allocation concealment: + Assessor blinding to outcome data: + Incomplete data addressed: + Selective reporting: ? Sample size calculated: +
Lavery 2004 [38]	*n* = 85 (I = 41, C = 44) Male: 49% Mean age: 55 years Previous ulcers: 41% T2DM: NR Mean diabetes duration: 14 years Ulcer risk: IWGDF risk group 2/3	Intervention: infrared thermometry and a complex intervention provided by treating physician (evaluation), nurse case manager (contact) and podiatrist (follow-up) Control: complex intervention; foot evaluation by a podiatrist every 10–12 weeks, therapeutic footwear, diabetic foot education	Footwear, education and professional foot care	Foot complications: ulcers, Charcot foot, infection and amputation (*n*) Quality of life: pre- and post-physical functioning, role physical, bodily pain, general health, vitality, social functioning, role emotional, mental health (SF-36 scores) Follow-up: 6 months	Sequence generation: ? Allocation concealment: ? Assessor blinding to outcome data: + Incomplete data addressed: + Selective reporting: + Sample size calculated: –
Lavery 2007 [39]	*n* = 173 (I1 = 59, I2 = 56, C = 58) Male: 54% Mean age: 65 years Previous ulcers: 100% T2DM: 95% Mean diabetes duration: 13 years Ulcer risk: high (10 g monofilament, VPT ≥25 V, palpation of pulses, Doppler, ankle brachial index ≥0.07)	Infrared thermometry and a complex intervention; study nurse for contact, treating physician for foot evaluations, podiatrist for assessing shoes/insoles I1: enhanced care with infrared thermometry I2: structured care with a structured daily foot self-inspection Control: standard care	Lower-extremity evaluation, education programme, therapeutic insoles and footwear All participants received a pedometer to record their daily activity in a log book Participants were told to inspect their feet daily and to contact a nurse if necessary	Foot ulcers (*n*), foot trauma, fracture, death, osteomyelitis, time to ulceration (days) Follow-up: 15 months	Sequence generation: + Allocation concealment: + Assessor blinding to outcome data: + Incomplete data addressed: + Selective reporting: + Sample size calculated: +
Skafjeld 2015 [40]	*n* = 41 (I = 21, C = 20) Male: 56% Mean age: 58 years Previous ulcers: 100% T2DM: 71% Mean diabetes duration: 18 years Ulcer risk: IWGDF risk group 3	Intervention: foot skin temperature monitoring, theory-based counselling provided by study nurse, contact study nurse if increase in temperature for >2 days Control: standard care	Foot care and recording observations daily, customised footwear	Ulcer (*n*), increased skin temperature (°C), customised footwear worn (h/day), contacts with study nurse Follow-up: 12 months	Sequence generation: + Allocation concealment: ? Assessor blinding to outcome data: + Incomplete data addressed: + Selective reporting: + Sample size calculated: –
Complex interventions					
Cisneros 2010 [41]	*n* = 53 (I = 30, C = 23) Male: 62% Mean age: 62 years Previous ulcers: 28% T2DM: 96% Mean diabetes duration: 14.5 years Ulcer risk: IWGDF risk group (I/C) 1 (6/10), 2 (15/7), 3 (3/3) or 4 (6/3)	Intervention: complex intervention Therapeutic education in groups of eight, 4× 90 min provided by researcher, two pairs of protective shoes, testing for neuropathy Control: information on regular foot care and footwear use according to spontaneous demand during individual consultations with the researcher	Routine care from staff, instructions on foot care when requested, testing for neuropathy	Ulcer occurrence (*n*), ulcer recurrence (*n*), time to foot ulceration (survival time – quarterly evaluations) Follow-up: 24 months Ulcerations were noted to occur more frequently in those at high risk	Sequence generation: ? Allocation concealment: ? Assessor blinding to outcome data: + Incomplete data addressed: ? Selective reporting: + Sample size calculated: –
LeMaster 2008 [42]	*n* = 79 (I = 41, C = 38) Male: 51% Mean age: 66 years Previous ulcers: 42% T2DM: 94% Mean diabetes duration: 11 years Ulcer risk: moderate or high risk	Intervention: complex intervention Part 1 (1–3 months): physical therapist led exercises to strengthen lower-extremity muscles and promote balance over eight sessions Part 2 (4–12 months): increase in moderately intense activity by 50% over 12 months among community-dwelling participants Provided by physical therapist and study nurse Control: standard care	Foot-related self-care skill education, daily foot examination Usual medical care from their own healthcare providers Participants were referred to orthotists or podiatrists for therapeutic footwear at enrolment	Foot ulcer rates (lesions/lesion episode, full-thickness ulcer/ulcer episode, weight-bearing full-thickness plantar ulcer/ulcer episode) (*n*) Step activity, person-years at risk Follow-up: 12 months	Sequence generation: + Allocation concealment: + Assessor blinding to outcome data: + Selective reporting: + Incomplete data addressed: + Sample size calculated: +
Liang 2012 [43]	*n* = 62 (I = 31, C = 31) Male: 56% Mean age: 56 years Previous ulcers: 0% T2DM: 87% Mean diabetes duration: 11 years Ulcer risk: ADA risk category 1/2/3 High risk, *n* = 100%	Intervention: complex intervention Foot care kit containing foot care cream, 10 g monofilament, thermometer to measure water temperature for washing feet, alcohol cotton pieces and a mirror Daily foot care and diabetes education classes provided by a diabetes nurse-led multidisciplinary team (three endocrinologists, four nurses and one dietitian) Control: standard care	Conventional care alone according to ADA standards; medication adjustment, foot assessment and 2 h of education about diabetes foot care	Ulcers (*n*, %), amputation (*n*, %), HbA_1c_ (%), diabetes knowledge, foot care behaviour Follow-up: 24 months	Sequence generation: ? Allocation concealment: ? Assessor blinding to outcome data: ? Selective reporting: ? Incomplete data addressed: + Sample size calculated: –
Litzelman 1993 [44]	*n* = 396 (I = 191, C = 205) Male: 19% Mean age: 60 years Previous ulcers: NR T2DM: 100% Mean diabetes duration: 10 years Ulcer risk: NR	Intervention: participant education sessions, self-foot care, reinforced through telephone follow-up (2 weeks) and postcard reminder (1 and 3 months) Informational flow sheets on foot-related risk factors for amputation in individuals with diabetes Prompts for healthcare providers to: (1) ask that participants remove their footwear; (2) perform foot examinations; and (3) provide foot care education Provided by nurse clinicians Control: care as usual plus standard care	1 year after the initial assessment, all participants underwent a repeated history and physical examination performed by nurse clinicians blind to participants’ randomised treatment	Participant outcomes: participant behaviour (scale) Behaviour of healthcare provider (%) Physical findings (ulcers, physical examination, dry/cracked skin, corns, calluses, ingrown nails, fungal infections, improperly trimmed nails, foot/leg cellulitis, leg deformity, sensory examination) (%) Follow-up: 12 months	Sequence generation: ? Allocation concealment: ? Assessor blinding to outcome data: + Selective reporting: + Incomplete data addressed: ? Sample size calculated: –
McCabe 1998 [45]	*n* = 1997 randomised (I = 997, C = 1000) Male: 53% Mean age: 60 years Previous ulcers: unclear T2DM: 80% Mean diabetes duration: NR Ulcer risk: low, moderate, high Ankle brachial index ≤0.75, history of foot ulcers = high risk	Intervention: primary foot screening examination with a biothesiometer and palpation of pedal pulses Foot pressures, subcutaneous oxygen levels, ankle brachial indices and X rays, weekly diabetic foot clinic for high-risk participants Provided by general diabetic outpatient clinic Control: participants were silently tagged and continued to attend the general outpatient clinic, but received no additional care	Participants were advised to inspect and wash their feet daily, avoid constricting clothing and footwear, wear prescribed footwear at all times and contact the clinic whenever they thought it necessary	Participant outcomes: ulcers (*n*), ulcer progressing to amputation (%) Process outcomes: screening cost (£), compliance with follow-up/treatment (%) Follow-up: 24 months	Sequence generation: ? Allocation concealment: ? Assessor blinding to outcome data: ? Selective reporting: + Incomplete data addressed: + Sample size calculated: –
Custom-made footwear and offloading					
Bus 2013 [46]	*n* = 171 (I = 85, C = 86) Male: 82.5% Mean age: 62 years Previous ulcers: 100% T2DM: 71% Mean diabetes duration: 17 years Ulcer risk: high (assessed with 10 g monofilament and vibration perception plus pedis tests)	Intervention: custom-made footwear, of which the offloading properties were improved and subsequently preserved based on in-shoe plantar pressure measurement and analysis A local specialist provided the footwear and a local orthopaedic shoe technician manufactured the footwear Control: custom-made footwear that did not undergo improvement based on in-shoe pressure measurement (i.e. usual care)	Each participant received written and verbal instructions on foot care and on proper use of footwear All footwear in both study groups was evaluated at delivery and at 3 month follow-up visits (pressure measurements, temperature monitor and activity monitor)	Ulcer recurrence (participants with ulcer, previous ulcer location, complicated foot ulcers); ulcer recurrence according to adherence and non-ulcerative lesions (all in *n*, %); in-shoe peak pressure, daily step count, adherence (mean ± SD) Follow-up: 18 months	Sequence generation: + Allocation concealment: + Assessor blinding to outcome data: + Incomplete data addressed: + Selective reporting: + Sample size calculated: +
Reiber 2002 [47]	*n* = 400 (I1 = 121, I2 = 119, C = 160) Male: 77% Mean age: 62 years Previous foot ulcers or infection requiring antibiotics: 100% T2DM: 93% Mean diabetes duration: <6 years: 33% 6–24 years: 11% ≥25 years: 56% Ulcer risk: high (assessed by 10 g monofilament and presence of foot deformity)	Therapeutic shoes with two types of inserts and standard care; provided by the study pedorthist provided and evaluated by a panel of three foot care specialists Intervention 1: three pairs of therapeutic shoes and customised medium-density cork inserts with a neoprene closed-cell cover Intervention 2: three pairs of therapeutic shoes and prefabricated, tapered polyurethane inserts with a brushed nylon cover Control: usual footwear and standard care	Participants continued to receive regular healthcare and foot care from the VA or GHC A lightweight terry-cloth house slipper (Tru-Stitch Footwear, Malone, NY, USA) with no internal seam and a textured sole was designed for all participants to use to minimise differences in out-of-shoe exposure	Lesions and ulcers (ulcers, non-ulcerative, total, person-years of follow-up); incidence per person (*n* participants with ≥1 ulcer, cumulative incidence per person, RR); incidence per person-year (ulcer and ulcer episode, *n*; incidence, RR); pivotal events for ulcer episodes (shoe and non-shoe related) (all in *n* and 95% CI) Follow-up: 24 months The majority of ulcers developed in those with foot insensitivity	Sequence generation: + Allocation concealment: ? Assessor blinding to outcome data: + Incomplete data addressed: + Selective reporting: + Sample size calculated: +
Rizzo 2012 [48]	*n* = 298 (I = 148, C = 150) Male: NR Mean age: 67 years Previous ulcers: 20% T2DM: 84% Mean diabetes duration: 18 years Ulcer risk: high (IWGDF risk group ≥2)	Intervention: orthoses and shoes, plus standard care Screening by an experienced podologist; foot and current ulcer risk evaluated by a team of a diabetologist, podologist, and orthopaedic technician Control: standard care	In-depth education on preventing ulceration, advice regarding footwear Urgent consultation within 24 h if ulcers developed	Foot ulcer (participants *n*), new foot ulcers (*n*), cumulative incidence of ulcers and recurrences (3 and 5 years; χ^2^, % and *p* value), ulcer due to trauma or hyperpressure (*n*, %), VPT (mean ± SD), cost evaluation (€) Follow-up: 12 months	Sequence generation: + Allocation concealment: ? Assessor blinding to outcome data: – Incomplete data addressed: + Selective reporting: + Sample size calculated: –
Lavery 2012 [49]	*n* = 299 (I = 149, C = 150) Male: 67% Mean age: 70.5 years Previous ulcers: 26.95% T2DM: NR Mean diabetes duration: 12.5 years Ulcer risk: high (IWGDF risk group 2/3)	Intervention: shear-reducing insole and complex intervention Concerns addressed by study nurse, evaluation conducted by a physician Control: standard care	Foot and lower-extremity evaluation by a physician every 10–12 weeks, education programme focused on foot complications and self-care practices Therapeutic shoes and standard insoles Contact with study nurse if concerned	Ulcers (*n*, %), footwear compliance (4, 4–8, 8–12, 12–16 h/day; *n*, %), time to ulcer (HR) Follow-up: 18 months	Sequence generation: ? Allocation concealment: ? Assessor blinding to outcome data: + Incomplete data addressed: + Selective reporting: + Sample size calculated: +
Ulbrecht 2014 [50]	*n* = 150 (I = 79, C = 71) Male: 68% Mean age: 59.5 years Previous ulcers: 100% T2DM: NR Mean diabetes duration: NR Ulcer risk: high (inability to feel 10 g monofilament, high plantar pressure, ankle brachial index)	Intervention: bespoke orthoses with offloading properties, provided by study coordinators (clinicians) Control: three different manufacturers’ orthoses plus three pairs of identical orthoses to be rotated while using the primary study footwear according to a written rotation protocol, changing the numbered orthoses in a set rotation every month; also offered one of two types of footwear models	Education on self-care behaviours with all participants, with a focus on wearing the study shoes for all steps taken and on examining the feet daily to note and report problems Educational brochure to reinforce advice	Ulcers (*n*, %), peak barefoot plantar pressure vs lesion (ulcer, non-ulcerative, no lesion; kPa) Questionnaires for quality of life (scaled to 100), foot self-care (0–1), fear of falling (scale to 100), participant satisfaction (five-point Likert scale) Follow-up: 1, 3 and 6 weeks, then every 3 months for another 15 months (potential 16.5 months)	Sequence generation: + Allocation concealment: + Assessor blinding to outcome data: + Incomplete data addressed: + Selective reporting: + Sample size calculated: +
Uccioli 1995 [51]	*n* = 69 (I = 33, C = 36) Male: 62% Mean age: 60 years Previous ulcers: 100% T2DM: 75% Mean diabetes duration: 17 years Ulcer risk: high (mean VPT ≥25 V)	Intervention: therapeutic shoes with custom insoles specially designed for individuals with diabetes (Podiabetes by Burrato Italy) Control: participants were free to wear ordinary shoes or their own non-therapeutic shoes unless clearly dangerous	All participants received the same educational guidelines on foot care and general information on the importance of appropriate footwear (i.e. proper size, durability and sole)	Ulcer relapses (*n*, %), cumulative incidence of relapse (multiple regression analysis), ulcer relapse between groups (χ^2^, %, *p* value), ulcer-free time, peripheral neuropathy (VPT, peripheral vascular disease), ankle brachial index (mean ± SD), use of therapeutic shoes Follow-up: 12 months	Sequence generation: ? Allocation concealment: ? Assessor blinding to outcome data: ? Incomplete data addressed: + Selective reporting: + Sample size calculated: –
Digital silicone devices					
Scirè 2009 [32]	*n* = 167 (I = 89, C = 78) Male: NR Mean age: 56.5 years Previous ulcers: unclear T2DM: 88% Mean diabetes duration: 16 years Ulcer risk: high (VPT ≥25 V)	Intervention: digital silicone orthoses (Podikon, Epitech, Saccolongo, Italy) and regular care at the diabetic foot clinic Control: no orthoses, but regular care at the diabetic foot clinic	Callus management; soft insole and extra-deep shoe	Ulcers (%), hyperkeratosis (plantar, dorsal, interdigital; %), skin hardness (%) Stable deformities (%) Podobarometric evaluation^b^ (pre- and post-evaluation in mean ± SD) Follow-up: 3 months	Sequence generation: + Allocation concealment: ? Assessor blinding to outcome data: + Incomplete data addressed: + Selective reporting: + Sample size calculated: –
Antifungal nail lacquer					
Armstrong 2005 [30]	*n* = 70 (I = 34, C = 36) Male: 97% Mean age: 70 years Previous ulcer: 57% T2DM: NR Mean diabetes duration: 12 years Ulcer risk: high (IWGDF risk group 2/3)	Intervention: antifungal treatment (ciclopirox 8%) and self-management (daily inspection) Control: self-management (daily inspection) A staff podiatrist examined each participant at recruitment A clinician familiar with the care and status of participants staffed a foot hotline 24 h/day	Preventative care programme and telephone support	Ulcers, unexpected visits, missed appointments, tinea pedis/ hyperkeratosis at start and end of study (%) Follow-up: 12 months	Sequence generation: + Allocation concealment: ? Assessor blinding to outcome data: ? Incomplete data addressed: + Selective reporting: + Sample size calculated: –
Elastic compression stockings					
Belcaro 1992 [31]	*n* = 160 (I = 80, C = 80) Male: 50% Mean age: 53 years Previous ulcers: none T2DM: NR Mean diabetes duration: 15 years Ulcer risk: microangiopathy measured with laser Doppler, VPT also measured	Intervention: knee elastic stockings with compression at the ankle of 25 mmHg, worn for at least 6 h/day while active and/or working Control: no stockings	NR	Ulcers (*n*, %), number of limbs (*n*) Deterioration of microcirculation Supine resting flux (mean ± SD) Venoarteriolar response (median and range) Follow-up: 48 months	Sequence generation: ? Allocation concealment: – Assessor blinding to outcome data: – Incomplete data addressed: + Selective reporting: + Sample size calculated: –
Podiatric care					
Plank 2003 [33]	*n* = 91 (I = 47, C = 44) Male: 56% Mean age: 65 years Previous ulcers: 100% T2DM: 93% Mean diabetes duration: 16 years Ulcer risk: high (reduced sensation assessed by 128 Hz tuning fork, 5.07 monofilament)	Intervention: chiropodist care and standard care Control: chiropodist care and standard care according to participant preference	Instruction on the possible benefits of regular chiropody care and the aim of the study	Ulcers (feet and participants), death, amputation (*n*, %). Follow-up: 12 months	Sequence generation: + Allocation concealment: + Assessor blinding to outcome data: ? Incomplete data addressed: + Selective reporting: + Sample size calculated: –

^a^Risk of bias: low (+), uncertain (?) or high (–)

^b^Includes total surface of the foot (cm^2^), average weight-bearing pressure (kPa), weight distribution compared with the total (%), weight distribution compared with the rear foot (%), static maximum peak pressure (kPa) and dynamic maximum peak pressure (kPa)

C, control; DFS-SF, Diabetic Foot Scale-Short Form; GHC, Group Health Co-operative; HADS, Hospital Anxiety and Depression Scale; I, intervention; IWGDF, International Working Group on the Diabetic Foot; NAFF, Nottingham Assessment of Functional Footcare; NR, not reported; T2DM, type 2 diabetes mellitus; VA, Veterans Affairs; VPT, vibration perception threshold

Overall, the included trials assessed eight different types of interventions to prevent foot ulceration, which we grouped as follows: (1) education alone (three trials) [34–36]; (2) dermal infrared thermometry (four trials) [37–40]; (3) complex interventions (five trials) [41–45]; (4) custom-made footwear and offloading insoles (six trials) [46–51]; (5) digital silicone device (one trial) [32]; (6) antifungal treatment (one trial) [30]; (7) elastic compression stockings (one trial) [31]; and (8) podiatric care (one trial) [33].

### Education alone

Three RCTs evaluated single-session education interventions of varying length and content for people at high risk of foot ulceration [34–36].

#### Meta-analysis

**Fig. 2:**
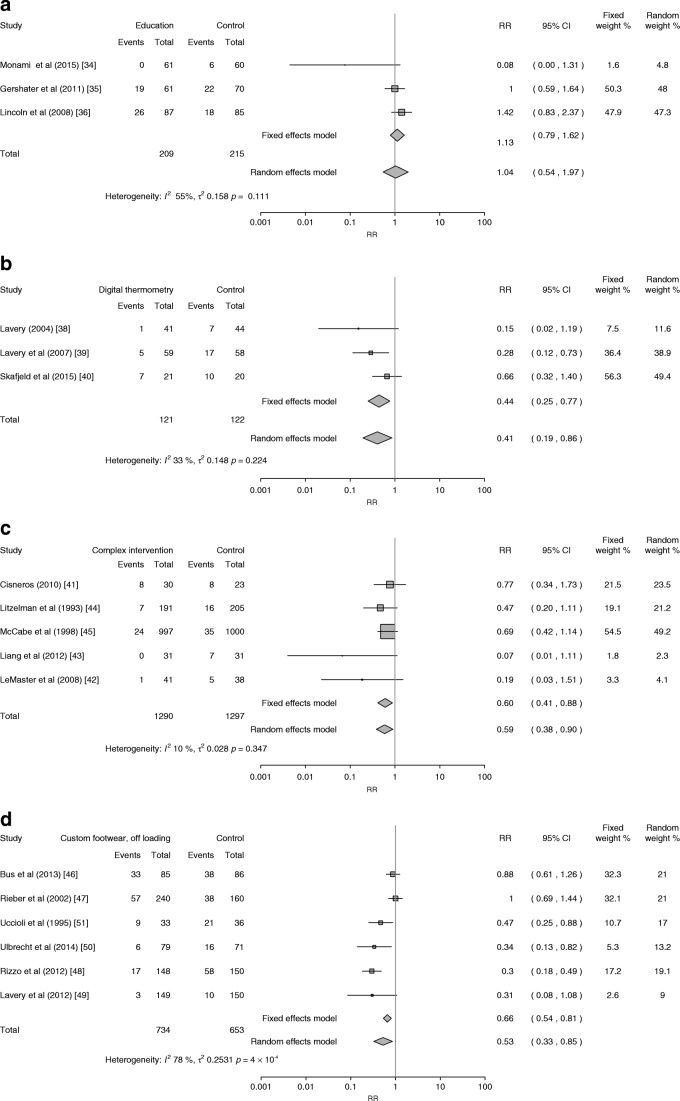
Forest plots of foot ulcers in people receiving standard care vs (**a**) education alone, (**b**) dermal infrared thermometry, (**c**) complex interventions and (**d**) custom-made footwear and offloading

(*n* = 423) (Fig. 2a) showed no statistically significant difference in the incidence of foot ulceration at 6 months compared with standard care and advice (RR 1.04 [95% CI 0.54, 1.97]) [34–36]. The quality of the included trials was variable, with only one trial [36] judged to be at low risk of bias across all domains. Other sources of potential bias arose from one trial [34] being stopped early and another [35] reporting an interim analysis before target recruitment was reached [52].

#### Secondary outcomes

Two trials of education interventions reported data on amputation [34, 36], mortality [34], knowledge [34], behaviour [36] and/or quality of life [36]. No amputations were recorded for participants in either arm at 6 months’ follow-up in one trial [34]. The other trial reported 3/85 amputations in the intervention arm vs 0/85 in the control arm at 6 months, and no difference (*n* = 9 in both arms) at 12 months [36].

One trial [34] reported that two participants, one in each arm, had died by 6 months. In the same trial, a statistically significant difference in knowledge (as measured by the Patient Interpretation of Neuropathy knowledge score) was observed in the intervention arm [34].

One trial [36] reported on quality of life and found no differences between the two arms on the Diabetic Foot Scale, but higher scores for those in the education arm on the Nottingham Assessment of Functional Footcare questionnaire, which assesses behaviour, compared with the control group.

### Dermal infrared thermometry

Four RCTs involving 468 participants with diabetes were identified [37–40]. In one trial [37], the numbers of participants randomised to either dermal infrared thermometry or standard care were not known, and so an RR and 95% CI could not be calculated.

#### Meta-analysis

A pooled analysis of data from three RCTs (*n* = 243) [38–40] found that dermal infrared thermometry reduced the number of foot ulcers in people with a history of foot ulceration (RR 0.41 [95% CI 0.19, 0.86]) (Fig. 2b). Outcomes were collected between 6 and 15 months. The quality of these trials was variable, with only one trial [39] judged to be at low risk of bias across all domains.

#### Secondary outcomes

Trials of dermal thermometry variously reported on amputation following infection [37], quality of life (36-item Short Form [SF-36]) [37], adherence to therapy [38, 39] and time to ulceration [39, 40].

In one trial, amputations following infections occurred in 0/41 participants in the intervention group vs 2/44 in the comparator group [38]. In the same trial there was no statistically significant difference in quality of life measured using SF-36 in any category or in the overall score [38].

Two trials [39, 40] found no statistically significant difference between the dermal thermometry group and the comparator group in the time that prescribed footwear and insoles were worn, as measured using a self-report questionnaire containing an ordinal scale of <4 to >12 h/day. The time to ulceration was statistically significantly longer in the dermal thermometry treatment group compared with standard care in one trial [39] but not in another [40].

### Complex interventions

Five RCTs evaluated the effects of complex interventions (i.e. integrated combinations of patient- or physician-level interventions and structural interventions) on the development of a foot ulcer [41–45].

#### Meta-analysis

A pooled analysis of data from five RCTs (*n* = 2587) showed that complex interventions statistically significantly reduced the number of foot ulcers (RR 0.59 [95% CI 0.38, 0.90]) at 1 or 2 year follow-up (Fig. 2c), with little evidence of statistical heterogeneity (*I*^2^ = 10%; Fig. 2c) despite the variety of interventions tested. However, with the exception of one trial [42], all had a high risk of bias and the validity of these data may be compromised. One trial gave no information about the participants’ risk category [44], while three included people with no history of foot ulceration [41,43]. One trial included people who were at low/moderate or high risk of developing a foot ulcer, found that 75% of ulcers occurred in people with higher levels of risk; for the highest risk category (category 4), 2/6 individuals in the intervention group and 2/3 individuals in the comparator group developed foot ulcers [41].

None of the individual trial results reached statistical significance and only one [42] reported an a priori sample size calculation; however, one trial [45] recruited everyone attending the foot care service.

#### Secondary outcomes

Amputation [43, 45], time to ulceration [41] and/or knowledge [43] were reported in three trials. In one trial [43] amputations occurred only in the control arm (2/31 vs 0/31 in the intervention arm), and in a second trial [45] there were fewer amputations in the intervention group (one major and six minor amputations) compared with the control group (12 major and 13 minor) [45]. The time to ulceration was shorter in the control group vs the intervention group in one trial, but this did not reach statistical significance [41].

In one trial participants’ knowledge about foot care, as measured using a diabetes knowledge questionnaire, was statistically significantly better in the intervention group compared with the control group [43].

### Custom-made footwear and offloading insoles

Six RCTs evaluated custom-made footwear and offloading insoles [46–51].

#### Meta-analysis

**Fig. 3:**
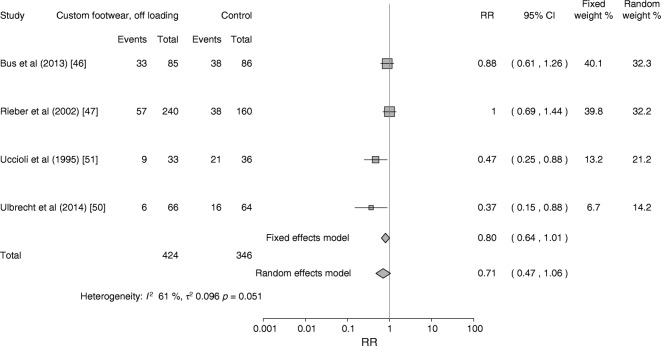
Subgroup analysis. Forest plot of foot ulcers in people with a history of foot ulceration receiving custom-made footwear and offloading vs standard care

A pooled estimate of data from six trials showed a beneficial association for custom-made footwear and offloading insoles on reducing the development of foot ulcers (pooled RR 0.53 [95% CI 0.33, 0.85]; Fig. 2d) for outcomes collected at 12–24 months in 1387 people, of whom 464 had no history of foot ulceration. There was evidence of considerable statistical heterogeneity (*I*^2^ = 78%), which we explored using baseline risk of ulceration in a subgroup analysis (Fig. 3). This pooled analysis of four trials [46, 47, 50, 51], all of which excluded people with no history of foot ulceration, failed to detect a statistically significant difference (RR 0.71 [95% CI 0.47, 1.06]). The six trials were of variable quality, with only two [46, 50] having a low risk of bias across all five domains.

#### Secondary outcomes

Adherence [46, 48, 49] and/or cost [48] data were reported in four trials. One trial measured adherence using a temperature-based monitor placed inside the shoe, and found that 35/85 participants in the intervention group and 42/86 in the control group adhered to wearing their allocated footwear [46]. The trial authors conducted a subgroup analysis in participants who wore their allocated footwear, which showed a statistically greater reduction in ulcer recurrence in the intervention group; however, the analysis using data from the entire trial population failed to detect a beneficial association. A second trial of custom-made footwear and offloading insoles measured adherence using a self-reported physical activity questionnaire, and found that footwear and insole use was high in the groups who received cork inserts (83%) and prefabricated insoles (86%) [47]. A third trial measured participant compliance with footwear using self-reports of the number of hours per day that the shoes were worn. There were no statistically significant differences between each group in the number of people who wore the shoes for less than 4 h per day (23/149 vs 16/150), 4–8 h (77/149 vs 83/150), 8–12 h (38/149 vs 46/150) and 12–16 h (10/149 vs 6/150) [49].

Cost data collected in one trial published in 2012 found the cost of supplying footwear and insoles to be €675 per person per year [48].

### Digital silicone devices

In one RCT of digital silicone devices [32], 167 participants with peripheral neuropathy, as defined by a vibration perception threshold of >25 V measured using a biothesiometer, and toe deformities (clawed toes, hallux valgus, interdigital lesions) were randomised to receive a bespoke silicone digital orthotic (*n* = 89) or standard care (*n* = 78). The number of ulcers was statistically significantly lower in the intervention group (RR 0.07 [95% CI 0.01, 0.55]) at 3 month follow-up. This trial had a low risk of bias in all domains except for allocation concealment, which was unclear.

### Antifungal treatment

In a trial of antifungal nail lacquer, participants in the intervention group (*n =* 34) received advice to inspect their feet daily and apply ciclopirox 8% to their toenails [30]. The control group (*n* = 36) received advice about daily foot inspections. A history of foot ulcers was reported by 57% of participants. After 12 months there were two ulcerations in each group (RR 1.06 [95% CI 0.19, 5.76]). The risk of bias was unclear in two domains: allocation concealment and blinding of the outcome assessor.

### Elastic compression stockings

An RCT of elastic stockings randomly allocated 160 people with no history of foot ulceration to either knee-length elastic stockings worn for 6 h/day or standard care [31]. There were three ulcers in the intervention group and ten in the control group, a difference that was not statistically significant (RR 0.37 [95% CI 0.11, 1.02]). The trial had a high or unclear risk of bias in the domains of sequence generation, allocation concealment and assessor blinding.

#### Secondary outcomes

Thirteen limbs were reported as lost during the 48 month trial; 3/74 in the intervention arm and 10/75 in the control arm.

### Podiatric care

One trial compared free chiropody care (*n* = 47) with no chiropody care (*n* = 44) for people all at high risk of foot ulceration [33]. Those receiving free chiropody were recommended to seek care at least once per month. The control group could seek chiropody if they were willing to pay for it, and their standard care included advice on the possible benefits of regular chiropody. There was no statistically significant difference in the number of ulcerations in the two groups (RR 0.67 [95% CI 0.43, 1.05]). This trial had a low risk of bias in all domains except assessor blinding to outcome data, which was unclear.

#### Secondary outcomes

There were 2/47 amputations in the intervention arm vs 1/44 in the control arm. Deaths were recorded as 2/47 in the intervention arm vs 4/44 in the control arm [33].

Data for other secondary outcomes of interest, such as gangrene, self-care, hospital admissions, timing of screening and adverse events or harms, were absent from the trial reports.

### Ongoing trials

The search for ongoing trials of foot ulcer prevention in diabetes from the ClinicalTrials.gov website found 24 studies being conducted worldwide. The stated interventions in these studies are: physiotherapy (*n* = 1), skin temperature (*n* = 6), hygiene (*n* = 1), offloading insoles (*n* = 10), risk stratification (*n* = 2), PET-CT (*n* = 1), amniotic tissue (*n* = 1) and unclear (*n* = 2). The list of these studies can be obtained from the corresponding author.

## Discussion

The purpose of this systematic review was to evaluate the evidence base and obtain summary statistics for preventative interventions for foot ulceration in diabetes to create a cost-effective, evidence-based care pathway. The meta-analyses of dermal infrared thermometry, complex interventions and therapeutic footwear with offloading insoles suggest that these interventions can help prevent foot ulceration in people with diabetes.

The meta-analysis of data from RCTs of dermal infrared thermometry in people with a history of foot ulceration and a moderate to high risk of ulceration indicates that this is a promising intervention deserving of further evaluation in randomised trials with larger participant samples, and we note from our search of the ClinicalTrials.gov trial registry that new trials are currently underway. If foot ulcer prevention can be confirmed in large, well-conducted trials, this form of self-monitoring could relieve pressure on healthcare systems. However, advising individuals to abstain from all weight-bearing activities when foot temperatures rise by more than 4°C may prove challenging, and poor adherence might diminish any benefit in a real-world context outside of a trial setting.

Specialist foot care, of the type evaluated in the included trials of complex interventions, is considered a marker of good-quality diabetes service delivery and it is intuitively correct to suppose it leads to improved outcomes. While a statistically significant reduction in foot ulcers was apparent in our meta-analysis, such an effect was not evident in any single trial. This does support the suggestion of others that very large sample sizes may be needed for trials of this nature [53]. Surprisingly, there was a low level of statistical heterogeneity in the pooled data, despite quite marked differences in the clinical care provided in the intervention arms of the trials and the participation of people with three different levels of ulcer risk.

Our review did not identify any trials of complex interventions that reflect the composition of multidisciplinary foot services as recommended in clinical guidelines [54–56]. These influential documents advise the involvement of diabetologists, podiatrists, vascular surgeons, diabetes specialist nurses and orthotists as the core team in a diabetes foot care service, but patient outcomes from such healthcare service arrangements have not been evaluated in RCTs. An evaluation of outcomes from people at different levels of ulceration risk who receive care in specialist foot care settings would be worthwhile.

The true value of therapeutic footwear and offloading insoles in preventing foot ulcers has been obscured by contradictory trial results and poor interpretation of data in systematic reviews; two larger trials involving only those with a history of foot ulcers both failed to detect evidence of effectiveness [46, 47], and visual inspection of our analyses of pooled data from all six trials shows greatest beneficial effect in those where the majority of participants were considered to be at high or moderate risk but had not experienced a foot ulcer [48, 49], albeit only one reached statistical significance [48]. Our subgroup analysis of data from four trials of participants with a history of foot ulceration found no statistically significant difference in the number of recurrent ulcers between the custom footwear and control groups.

This observation calls into question the conclusions of other systematic reviews evaluating footwear and insoles in the prevention of foot ulcers [6, 17, 24]. The most recent included randomised and non-randomised data and adopted a consensus approach to the analysis. The reviewers concluded that: ‘The evidence base to support the use of specific self-management and footwear interventions for the prevention of recurrent plantar foot ulcers is quite strong, but…is practically non-existent for the prevention of a first foot ulcer and non-plantar foot ulcer’ [24]. An individual participant data analysis using data from these six trials together with data from the ten ongoing studies of offloading insoles identified by our search of the ClinicalTrials.gov database could permit subgroup analyses to explore the value of footwear and offloading insoles in people with different baseline risks, and potentially resolve these ongoing uncertainties.

The marked reduction in ulcerations reported with the use of a dermal silicone device by individuals at high risk of ulceration is encouraging [32]. These devices are simple to make at the chair-side and easy for wearers to keep clean. Although they are a type of offloading intervention, we did not include these data in the meta-analysis of footwear and offloading insoles because they differ substantially in that they are only worn around the toes.

Three separate small trials [30, 31, 33] evaluating, respectively, the effects of a daily application of a fungal nail lacquer (ciclopirox 8%) with daily foot inspections, the use of elastic compression stockings and podiatry all failed to show a reduction in foot ulcers, possibly as a result of small sample sizes.

### Strengths and limitations of this review

We have comprehensively reviewed a body of evidence from RCTs and made the fullest use of the data currently available to derive best estimates of treatment effects to inform a wider piece of work. In so doing we have highlighted uncertainties, gaps and limitations in the existing evidence base to inform practice, generated new research hypotheses and added value to this area of research.

The weaknesses of this review arise from the potential biases identified in many of the trial reports, especially for complex interventions, which may have produced unreliable results. Previous authors of systematic reviews have cited a lack of similarity between studies [13], lack of standardisation in terminology, prescription, manufacture and material properties of interventions [16], heterogeneity in study designs, methodology and participant populations [18], and differences in participant demographics [22] as reasons for not conducting meta-analyses, and we are aware of the potential limitations in the pooled analyses that we present here, both in the number and quality of trials. We have tried to produce conservative, less biased summary measures by adopting an intention-to-treat approach and a random-effects model. We acknowledge criticisms about the use of the latter [57], but believe the insights gleaned and the generation of new research hypotheses justifies our decision to pool data [58].

### Conclusions

Our analyses found evidence of beneficial effects for four types of interventions used to prevent foot ulcers in people with diabetes, but considerable uncertainty remains about what works and who is most likely to benefit. Attention should be given to recommendations for the conduct of trials of interventions for the foot in diabetes, and researchers conducting future trials should endeavour to complete the trial to target recruitment as informed by an a priori sample size calculation [29, 59].

## Electronic supplementary material

ESM (PDF 407 kb)

## Data Availability

A copy of the extracted dataset can be obtained from the corresponding author.
