# Supplementary material for: Preventing foot ulceration in diabetes: systematic review and meta-analyses of RCT data
Source: Diabetologia. 2019 Nov 27;63(1):49–64. doi: 10.1007/s00125-019-05020-7 (PMC6890632; doi:10.1007/s00125-019-05020-7)
Supplement: Supplementary file 1 — (PDF 407 kb) [file 125_2019_5020_MOESM1_ESM.pdf]

ESM table 1. Medline Search

#	Searches
1.	MeSH descriptor: [Foot Orthoses] explode all trees
2.	MeSH descriptor: [Shoes] explode all trees
3.	MeSH descriptor: [Health Education] explode all trees
4.	MeSH descriptor: [Primary Health Care] explode all trees
5.	MeSH descriptor: [Emollients] explode all trees
6.	insole*
7.	footwear*
8.	educat*
9.	specialist car*
10.	multi disciplinary team*
11.	multidisciplinary team*
12.	routine podiatry car*
13.	MeSH descriptor: [General Practice] explode all trees
14.	MeSH descriptor: [Community Health Services] explode all trees
15.	off load*
16.	offload*
17.	emollient*
18.	shoe*
19.	{or #1-#18}
20.	MeSH descriptor: [Foot] explode all trees
21.	MeSH descriptor: [Foot Diseases] explode all trees

22.	MeSH descriptor: [Diabetic Foot] explode all trees
23.	MeSH descriptor: [Diabetic Neuropathies] explode all trees
24.	MeSH descriptor: [Diabetes Mellitus] explode all trees
25.	MeSH descriptor: [Diabetic Angiopathies] explode all trees
26.	MeSH descriptor: [Diabetes Complications] explode all trees
27.	MeSH descriptor: [Podiatry] explode all trees
28.	MeSH descriptor: [Foot Ulcer] explode all trees
29.	MeSH descriptor: [Skin Ulcer] explode all trees
30.	MeSH descriptor: [Ischemia] explode all trees
31.	MeSH descriptor: [Bacterial Infections] explode all trees
32.	diabet* near/3 ulcer*
33.	diabet* near/3 (foot or feet)
34.	diabet* near/3 wound*
35.	diabet* near/3 amputat*
36.	{or #20-#35}
37.	#19 and #36
